# Elevated MFG-E8 in CSF in the Early Stage Indicates Rapid Recovery of Mild Aneurysmal SAH Patients

**DOI:** 10.1155/2022/6731286

**Published:** 2022-10-11

**Authors:** Cong Pang, Zheng Peng, Xiaojian Li, Yongyue Gao, Xunzhi Liu, Han Wang, Yue Lu, Zong Zhuang, Qingrong Zhang, Wei Li, Chunhua Hang

**Affiliations:** Department of Neurosurgery, Affiliated Drum Tower Hospital, Medical School of Nanjing University, Jiangsu, China

## Abstract

**Background:**

Aneurysmal subarachnoid hemorrhage (aSAH) can impair blood perfusion in brain tissue and cause adverse effects. Microglia, which are the inherent immune cells of the brain, significantly activate and play a role in phagocytosis, anti-inflammatory, proinflammatory, and damage repair in this process. Milk fat globule epidermal growth factor 8 (MFG-E8) is the bridging molecule of this process and mediates the activation and biological effects of microglia.

**Methods:**

We obtained cerebrospinal fluid (CSF) from patients with aSAH at various times (the third day, seventh day, and ninth day) as well as from patients in the control cohort. MFG-E8 protein levels in CSF were measured by enzyme-linked immunosorbent assay (ELISA). Meanwhile, we evaluated the GCS and GOS of aSAH patients on admission and on the third day, seventh day, ninth day, and at discharge. Then, we analyzed the association between the levels of MFG-E8 and the changes in GCS and GOS.

**Results:**

MFG-E8 expression rose in the early stage on the third day and reached equilibrium around day 7 and day 9. The levels of MFG-E8 on the third day were associated with the change in GOS on the seventh day (*r* = 0.644, *p* = 0.018) and ninth day (*r* = 0.572, *p* = 0.041) compared with admission but were not correlated with the change on day 3 or at discharge. The levels of MFG-E8 were not correlated with any change in GCS.

**Conclusions:**

We found that aSAH resulted in an upregulation of MFG-E8 in CSF. Moreover, high MFG-E8 levels in the early stage indicated a rapid recovery of mild aSAH patients.

## 1. Introduction

Subarachnoid hemorrhage (SAH) is a type of blood oozing from damaged vessels into the subarachnoid space because of different kinds of brain damage. It is a destructive cerebrovascular disease that affects the cerebral blood perfusion state [1] and contributes to cerebral vasospasm [2], early brain injury [3], chronic cerebral ischemia [4], and various systemic complications [5]. These injuries often lead to a poor prognosis [6]. Among them, aneurysmal subarachnoid hemorrhage (aSAH) accounts for 85% of spontaneous SAH, and the mortality rate has reached 50% [7–9]. However, with the advancement of current treatment, the mortality and disability rates of aSAH are still high [10]. Therefore, it is increasingly important to explore the pathophysiology of aSAH, which will help the clinical treatment of aSAH.

According to relevant reports, as inherent immune cells in brain tissue, microglia are activated when aSAH occurs. In this process, microglia release a series of cytokines, mediate the repair of damaged neurons, maintain the balance between proinflammatory and anti-inflammatory activities, activate phagocytosis, and so on [11, 12]. It performs an integral function in the repair of SAH [13].

Milk fat globule epidermal growth factor 8 (MFG-E8) is a key cytokine that is secreted by phagocytes and mediates phagocytosis. As a bridging molecule among cells [14], it can promote macrophages to exert a series of biological effects, such as anti-inflammation and phagocytosis, and is also expressed in breast cells and endothelial cells [15]. Several research reports have illustrated that MFG-E8 is involved in a number of important physiological and pathological processes. For example, MFG-E8 expression is related to the downregulation of the inflammatory response in diabetes mellitus [16]. In the injury response, MFG-E8 expression can promote angiogenesis and the healing of skin wounds [17], and it also performs a crucial function in atherosclerosis [18] and autoimmune diseases [19]. Microglia are macrophages in the central nervous system (CNS). Some acute and chronic nerve injuries can trigger inflammatory responses and activate microglia that secrete MFG-E8 [20]. Activated microglia might play a protective role toward nerve cells by regulating cell apoptosis, oxidative stress, and the inflammatory response [15].

When aSAH occurs, microglia can be activated. As a bridging molecule, MFG-E8 is secreted to mediate a series of biological functions of microglia [21]. This process has been confirmed in animal models [15]. However, there has been little concern about the alterations in the levels of MFG-E8 in aSAH patients until now. Hence, in the current research, we aimed to observe the dynamic alterations of MFG-E8 in patients' cerebrospinal fluid (CSF) at different times after aSAH and tried to explore the correlation between MFG-E8 levels and the outcomes of aSAH patients.

## 2. Materials and Methods

All of the procedures conducted in human studies were designed in strict accordance with the *Declaration of Helsinki* and authorized by the medical institutional review board (No. 2020-041-01) at Affiliated Drum Tower Hospital, Medical School of Nanjing University. All clinical samples were acquired with the consent of the patients included in the present study.

### 2.1. Selection of Patients

The following guidelines were used to select the experimental cohort: (1) Hunt-Hess grade I or II, (2) no other CNS disorders, (3) within 2 days following aSAH, individuals in the experimental cohort were hospitalized and underwent interventional treatment, and (4) no connective tissue illnesses, malignant tumors, diabetes, or other systemic diseases.  Table 1 shows the health status of the patients in the experimental cohort. The control cohort comprised individuals who required lumbar subdural anesthesia for surgical procedures and did not have SAH or any other CNS condition.

### 2.2. Identification and Collection of Samples

The CSF samples used in the present study were obtained from both the experimental cohort (*n* = 14) and the control cohort (*n* = 11). The CSF samples of the experimental cohort were collected from patients on the third, seventh, and ninth days following aSAH. Additionally, the CSF samples obtained from patients in the control cohort were utilized as normal controls. A sterile tube was used to centrifuge all clinical samples (3000 g, 5 min), and the supernatant was then collected and kept at -80 degrees Celsius.

After we acquired enough samples, we performed enzyme-linked immunosorbent assay (ELISA) to evaluate the MFG-E8 levels in the CSF. We utilized a commercial human ELISA kit (ab235638, Abcam, Shanghai, China) for this experiment according to the instructions stipulated by the manufacturer. Specifically, we started by equilibrating the samples and reagents and placing them at ambient temperature and setting up the blank well, control well, and sample well. The control well was supplied with 50 *μ*l of the standard sample. We introduced 40 *μ*l of sample dilution and 10 *μ*l of sample into the sample well and subsequently mixed the samples together. Incubation was performed for 30 minutes at 37 degrees Celsius after the plate had been sealed. After sealing the plate, we dumped all of the liquid, added the washing liquid after drying, and rinsed it for 30 seconds five times. Each well, with the exception of the blank well, received 50 *μ*l of enzyme-labeled reagent. Following incubation and rinsing (using the same procedures as before), we introduced the developer, stirred the wells, and allowed the color to develop for 15 minutes at 37°C in the dark. The reaction was stopped when the microplate became blue by adding 50 *μ*l of stop buffer. After that, the microplate began to change to a yellow color. The optical density (OD) of each well was determined at a wavelength of 450 nm. Then, we generated a standard curve, setting the concentrations as the abscissa and the values of OD as the ordinates. Subsequently, we obtained the regression equation of the standard curve. Finally, we computed the MFG-E8 levels using the actual OD and dilution values obtained from the samples.

### 2.3. Evaluation and Categorization of Patients Based on Their Recovery Status

**Figure 1:**
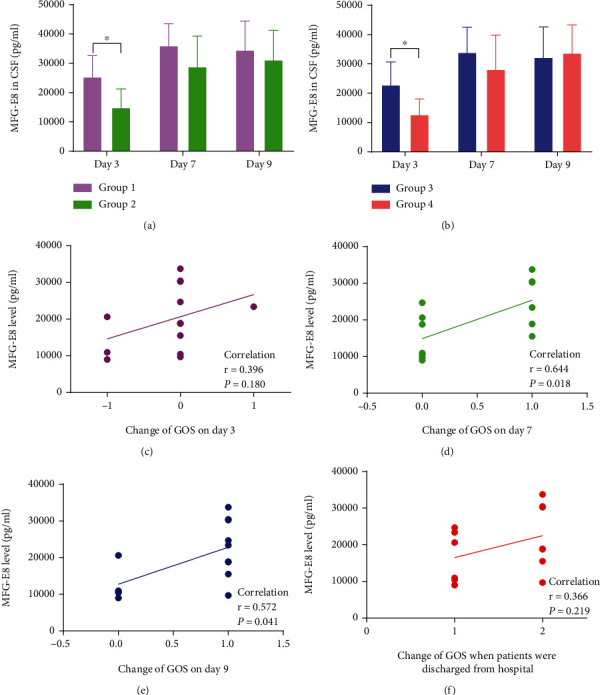
The relationship between MFG-E8 and changes in GOS. (a) The difference in MFG-E8 levels between cohort 1 and cohort 2 (cohort 1: GOS with elevation on day 7 in contrast with admission and cohort 2: GOS with no elevation on day 7 in contrast with admission). (b) The difference in MFG-E8 levels between cohort 3 and cohort 4 (cohort 3: GOS with elevation on day 9 compared with admission and cohort 4: GOS with no elevation on day 9 compared with admission). (c–f) The correlation between the levels of MFG-E8 on day 3 and the alterations in GOS scores at distinct times following aSAH. ^∗^*p* < 0.05 between cohorts.

We examined the patients' GCS and GOS on the day of admission, the third day, the seventh day, the ninth day, and at discharge. In this study, we conducted bedside scores for all patients. All clinical scores are scored by a single person throughout the process to ensure the consistency of the test. Within two days following aSAH, all patients were hospitalized and underwent intervention treatment. As a result, the GOS and GCS scores obtained upon admission were employed as the baseline status. The GOS of the patients at the time of discharge reached the full score (as shown in Table 2). For the GOS, we excluded patients with a score of 5. We grouped the patients according to whether GOS had increased compared with admission and compared the expression levels of MFG-E8 between the cohorts. Only one patient had an elevated GOS score on the third day, and all patients' GOS was elevated upon discharge. As a result, we could not group the patients on day 3 and at discharge because of the sample size. We evaluated whether there was an increase between day 7 and admission (cohort 1 with elevation and cohort 2 with no elevation), as well as that between day 9 and admission (cohort 3 with elevation and cohort 4 with no elevation). Then, we analyzed the difference in MFG-E8 levels between cohorts. As shown in Figures 1(a) and 1(b), the levels of MFG-E8 on the third varied substantially between the cohorts. We defined the rising period of MFG-E8 as an early stage. Then, we analyzed the association between the levels of MFG-E8 on day 3 and the changes in GOS after aSAH.

For the GCS scores, we excluded patients with a score of 15 each time. As illustrated in Table 3, only one patient's score did not increase on day 3 compared to admission, and all of them reached the full score on day 7. The patient's GCS scores were the same after day 7 following aSAH. We could not divide patients according to whether there was an increase in GCS compared with admission. Considering that MFG-E8 on day 3 might play a biological role, we analyzed the correlation between the levels of MFG-E8 on the third day and the changes in GCS on the third day and seventh day.

### 2.4. Data Analysis

Analysis of the data was conducted utilizing GraphPad Prism 7.0 and SPSS 24.0. An analysis of differences in continuous variables across cohorts was performed utilizing the unpaired Student's *t* test. In this case, *p* < 0.05 indicated a significant difference. Data were presented as the mean ± SD. When evaluating the simple correlation among continuous variables, the Pearson correlation coefficient was employed. Therefore, the Pearson correlation coefficient was utilized to assess the relationship between the MFG-E8 levels on the third day and the alterations in GOS and GCS at various time points following aSAH.

## 3. Results

### 3.1. Changes of MFG-E8 Levels after aSAH

**Figure 2:**
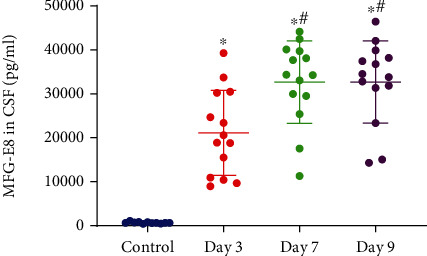
Changes in MFG-E8 levels in CSF measured by ELISA. The MFG-E8 protein levels at different times after aSAH and in the control cohort. ^∗^*p* < 0.05 compared with the control cohort; ^#^*p* < 0.05 compared with the day 3 cohort.

We believed that the control cohort could reflect the normal level of cytokines in CSF. As illustrated in Figure 2, the patients' MFG-E8 levels in CSF were relatively low in the control cohort, and a considerable elevation in the MFG-E8 levels was observed on day 3 after aSAH (*p* < 0.05). The levels of MFG-E8 reached equilibrium around day 7 and day 9, which remained elevated as opposed to that of the control cohort (*p* < 0.05). The difference between day 7 and day 9 was not significant.

### 3.2. Relationship between MFG-E8 and the Changes of GOS

The GOS scores of all patients at different times are recorded in Table 2. The GOS of each patient at discharge was full marks, indicating that the patients recovered well. Excluding patients who scored full marks each time, we grouped the patients according to the alterations in GOS on the seventh day and ninth day. On the seventh and ninth days, the MFG-E8 level on the third day was considerably enhanced in the cohort with an elevated GOS score in contrast with the cohort without elevation (*p* < 0.05). This validated our conjecture that the level of MFG-E8 could perform a crucial biological function in the early stage. The correlation was evaluated between the MFG-E8 level on the third day and the changes in GOS on the third day (*r* = 0.396, *p* = 0.180), seventh day (*r* = 0.644, *p* = 0.018), ninth day (*r* = 0.572, *p* = 0.041), and at discharge (*r* = 0.366, *p* = 0.219). The MFG-E8 level on the third day was strongly associated with the changes in GOS on day 7 and generally correlated with the changes on day 9 but not correlated with the changes on day 3 or at discharge.

### 3.3. Relationship between MFG-E8 and the Changes of GCS

**Figure 3:**
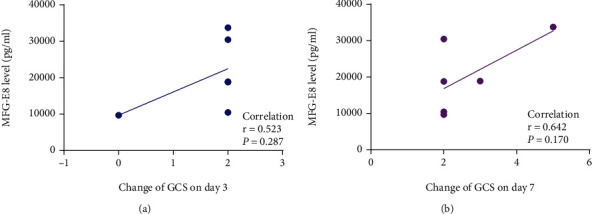
The link between MFG-E8 and the changes of GCS. (a) The association between MFG-E8 levels and changes in GCS on the third day. (b) The association between MFG-E8 levels and alterations in GCS on the seventh day.

The GCS scores of all patients at different times are recorded in Table 3. On admission, 8 out of 15 patients exhibited a GCS score of 15, with a minimum score of 10. All patients reached a full score of 15 on day 7, indicating that the patients' condition was mild and recovered well when discharged. Excluding patients whose scores were 15 each time, we evaluated the correlation between MFG-E8 levels on the third day and the alterations in GCS on the third day and seventh day and found that there was no statistical correlation (as shown in Figure 3).

## 4. Discussion

When cells are apoptotic, phosphatidylserine (PS), located in the inner layer of the cell membrane, will turn outward and appear in the outer layer of the cell membrane. This is a common biological process [22]. MFG-E8 is a bridging molecule that facilitates the biological effects of microglia. When different kinds of brain damage cause apoptosis of neuronal cells, MFG-E8 binds to PS, integrin *α*_v_*β*3/*α*_v_*β*5 [23], and vitronectin receptor [20]. Then, microglia can phagocytose apoptotic cells through the mediating effects of MFG-E8. Moreover, MFG-E8 can also inhibit apoptosis of neurons through the downstream FAK/PI3K/AKT pathway [15, 24]. The levels of MFG-E8 in CSF increased significantly on day 3 and reached equilibrium on day 7 after aSAH, which indicated that MFG-E8 did gradually increase and mediated certain biological effects after the occurrence of aSAH in patients. MFG-E8 is mainly secreted by microglia in the brain and can mediate related activities, which indicates that microglia can be constantly activated and play important biological roles in the process of aSAH.

In our current research, we found that MFG-E8 increased on day 3, indicating that it might begin to play a biological role in the early stage after aSAH. However, the effects required time to accumulate and were not immediately reflected on day 3. Therefore, MFG-E8 was not correlated with the changes in GOS on day 3 after aSAH. The levels of MFG-E8 on day 3 had a strong correlation with the changes in the GOS scores on the seventh day and were generally associated with the changes in GOS on the ninth day. This result indicated that the protective impacts of MFG-E8 in the early stage might be reflected on day 7, and the protective effect was the strongest. Although the positive effect of MFG-E8 could also be observed on day 9, this effect was weakened, and the correlation with the change in GOS decreased. On discharge, the positive effect weakened further, and the MFG-E8 levels on the third day were not associated with the changes in GOS. Therefore, the increase in MFG-E8 in the early stage brought time-effective effects, which might promote the patients' rapid recovery following aSAH.

Although the levels of MFG-E8 can affect the changes in GOS, we did not find a similar effect on the GCS scores. Most of the GCS scores were 15 or reached 15 quickly, resulting in a small sample size that can be selected for statistics. In addition, we selected low-grade aSAH patients. If they had high-grade aSAH, GCS might present a larger difference after aSAH. In the current experiment, the GCS of low-grade aSAH patients increased rapidly. As a result, we speculated that positive effects of MFG-E8 might be observed if we increased the observation frequency of GCS in the early stage, which will be our future research directions. Moreover, GCS evaluates the patient's eye status, speech response, and limb movements, which has certain limitations. Compared with GOS, GCS focuses more on evaluating the condition than prognosis. In short, the level of MFG-E8 in the early stage may highly suggest the rapid recovery of patients with aSAH.

In the present research, we discovered that when aSAH occurred, the MFG-E8 levels in the CSF increased and then reached a plateau. MFG-E8 may perform a protective function in the early stage. Although it did not change the clinical outcomes of aSAH patients, it might promote rapid recovery and bring positive effects to mild aSAH patients.

To our knowledge, this is the first study regarding the changes and possible effects of MFG-E8 in CSF in aSAH patients. We consider that MFG-E8 is mainly secreted by microglia, which are mainly found in the nervous system. Compared with examining changes in MFG-E8 in blood, detecting changes in CSF would be more intuitive [25]. However, the limits of our study are also obvious. The sample size was small. We found the expression pattern of MFG-E8 until day 9 after aSAH, but we lacked follow-up studies to determine how MFG-E8 changed subsequently. We could also conduct a posthospital visit to determine how MFG-E8 affects the long-term prognosis of patients [26]. We will improve these defects in future research. In this research, we did find that high levels of MFG-E8 contribute to rapid recovery in mild patients. The reason for not including the Hunt-Hess score of 3-5 is that these patients have severe clinical symptoms and may undergo craniotomy, which may affect the microenvironment of the patient's central nervous system and the MFG-E8 levels. It may result in inconsistent patient baseline levels. In addition, for severe patients, we want to minimize lumbar puncture procedures to reduce the impact on their own status. However, for mild patients, lumbar puncture is not only an examination but also a treatment. Hemoglobin that dissolved in CSF can be excreted while the CSF is obtained.

## 5. Conclusions

As a bridging molecule, MFG-E8 can mediate the interaction among microglia and other cells so that microglia can perform a crucial function in damage repair and neuroprotection [11]. In this experiment, we observed changes in MFG-E8 levels in the CSF of 14 patients with aSAH and 11 control patients. We found that MFG-E8 in CSF gradually increased and then reached a plateau, indicating that microglia could be activated and play a biological role in patients after aSAH. High levels of MFG-E8 in the early stage highly suggested a rapid recovery of mild aSAH patients, which might bring new approaches for clinical treatments of aSAH.

## Figures and Tables

**Table 1:** The health status of the patients in the experimental cohort.

Patient injury status						
Case	Gender	Age	Aneurysm location	Hunt-Hess	Admission GCS score	Admission GOS score
1	Male	42	V4 segment of left vertebral artery	II	13	3
2	Female	57	Anterior communicating artery	II	15	3
3	Female	57	Right posterior communicating artery	II	13	3
4	Male	49	The bifurcation of right middle cerebral artery	I	15	4
5	Female	51	Anterior communicating artery	II	10	3
6	Female	76	Anterior communicating artery	I	15	4
7	Female	46	Posterior communicating artery	II	12	3
8	Female	48	Apex of basilar artery	II	15	3
9	Female	65	Left anterior choroidal artery	I	15	4
10	Male	52	The bifurcation of middle cerebral artery	I	15	4
11	Female	65	Left posterior communicating artery	I	15	5
12	Female	49	Right posterior communicating artery	I	13	4
13	Male	68	Right anterior choroidal artery	II	13	3
14	Female	45	Apex of basilar artery	I	15	4

**Table 2:** The GOS scores of all patients at different times.

GOS scores of patients at different times					
Case	Admission GOS score	Day 3 GOS	Day 7 GOS	Day 9 GOS	GOS at discharge
1	3	3	3	4	5
2	3	3	4	4	5
3	3	3	3	4	5
4	4	3	4	4	5
5	3	3	4	4	5
6	4	3	4	4	5
7	3	3	4	4	5
8	3	3	4	4	5
9	4	3	4	4	5
10	4	4	4	5	5
11	5	5	5	5	5
12	4	4	4	4	5
13	3	3	4	4	5
14	4	5	5	5	5

**Table 3:** The GCS scores of all patients at different times.

GCS scores of patients at different times					
Case	Admission GCS score	Day 3 GCS	Day 7 GCS	Day 9 GCS	GCS at discharge
1	13	13	15	15	15
2	15	15	15	15	15
3	13	15	15	15	15
4	15	15	15	15	15
5	10	12	15	15	15
6	15	15	15	15	15
7	12	14	15	15	15
8	15	15	15	15	15
9	15	15	15	15	15
10	15	15	15	15	15
11	15	15	15	15	15
12	13	15	15	15	15
13	13	15	15	15	15
14	15	15	15	15	15

## Data Availability

The data used to support the findings of this study are available from the manuscript.
